# The association between new inflammation markers and frequent peritoneal dialysis-associated peritonitis

**DOI:** 10.1186/s12882-024-03496-z

**Published:** 2024-03-05

**Authors:** Jing Tang, Dongxue Wang, Yun Chen, Jinhong Feng

**Affiliations:** 1 Department of Nephrology, The Affiliated Wuxi People’s Hospital of Nanjing Medical University, Wuxi People’s Hospital, Wuxi Medical Center, Nanjing Medical University, Wuxi, China; 2 Department of Nephrology, Affiliated Hospital of Xuzhou Medical University, Xuzhou, China; 3 Department of Rheumatology, Affiliated Hospital of Xuzhou Medical University, Xuzhou, China

**Keywords:** Peritoneal dialysis-associated peritonitis, Systemic immune-inflammation index, C-reactive protein, High-density lipoprotein cholesterol

## Abstract

**Objective:**

To validate an association between new inflammation and frequent peritoneal dialysis-associated peritonitis (PDAP).

**Materials and methods:**

In China, retrospective clinical data were collected on 208 patients who received continuous ambulatory peritoneal dialysis (CAPD) between 2010 and 2021. The patients were divided into two groups: non-frequent PDAP (the interval between two peritonitis episodes of more than one year) and frequent PDAP (the interval between two peritonitis episodes of less than one year). Patients with their first episode of peritonitis had their age, gender, history of hypertension, diabetic disease, underlying renal disease, bacterial infection, and laboratory data collected. The outcomes of bacterial dispersion, systemic immune-inflammation index (SII), high-density lipoprotein cholesterol (HDL-C), C-reactive protein (CRP), and risk variables associated with frequent PDAP were analyzed.

**Results:**

There are differences between the two groups in dialysis time (*p* = 0.006), hypertensive nephropathy (*p* = 0.038), staphylococcus (*p* = 0.035), white blood cells (*p* = 0.001), neutrophil (*p* < 0.01), lymphocyte (*p* < 0.01), platelet(*p* = 0.01), SII(*p* < 0.01), CRP/HDL-C (*p* = 0.002), CRP (*p* < 0.001), serum creatinine (*p* = 0.007), blood urea nitrogen (*p* = 0.05), serum magnesium (0.03), serum potassium (*p* = 0.007), and dialysate polymorphonuclear cells (*p* = 0.004). Multifactorial logistic regression analysis found that SII (*p* < 0.001), CRP/HDL-C (*p* = 0.041), and Diabetes mellitus (*p* = 0.027) were independent risk factors for frequent PDAP. The ROC curve analysis revealed that combining SII with CRP/HDL-C resulted in the largest AUC area (AUC = 0.814).

**Conclusions:**

Our findings offer clinical proof of the combination of SII and CRP/HDL-C in patients with frequent PDAP.

## Introduction

Peritoneal Dialysis (PD) has become one of the primary renal replacement therapies for patients with end-stage renal disease (ESRD) due to the benefits of preserving residual renal function and the ease of promotion. Given the rapid global spread of coronavirus 2019 (COVID-19), PD outperforms hemodialysis (HD) as an effective renal replacement therapy with a lower risk of bacterial illness [1]. However, the problem of PDAP (peritoneal dialysis-associated peritonitis) is becoming more prevalent in clinical practice. PDAP is a major complication of PD patients that can account for more than 15% of patient deaths [2–4]. Furthermore, peritoneal ultrafiltration capacity is reduced after a single episode of severe peritonitis or multiple episodes of peritonitis. They could even cause sclerosing encapsulated peritonitis. They are essential contributors to PD patients converting to long-term hemodialysis [5–6]. Frequent episodes of PDAP cause peritoneal fibrosis, which leads to peritoneal function loss, decreased residual renal function, and increased rates of extubation and withdrawal in PD patients [7]. A cohort study in Taiwan found an increase in adverse outcome events in PD patients as the times of peritonitis increased [8].

The relevance of inflammation in PD patients is noteworthy. Numerous well-known inflammatory markers, such as neutrophils, lymphocytes, platelet count, and CRP, have been linked to PDAP [9–11]. However, focusing on just one marker is ineffective because these biomarkers are constantly influenced. The systemic immune-inflammation index (SII) has recently been advocated as a convenient and comprehensive marker. SII is an emerging indicator of inflammation that can comprehensively reflect the inflammatory and immune status of the body. SII is the ratio of neutrophil count × platelet count/lymphocyte count, which has been shown to be an independent predictor of tumor recurrence metastasis and prognosis [12]. CRP is a pattern recognition molecule in inflammation processing [13]. PD patients with altered nutritional status and chronic inflammation are frequently combined with dyslipidemia. According to some studies, HDL-C in ESRD patients is less effective at promoting cholesterol efflux and has antioxidant and anti-inflammatory deficiencies. In uremic patients, HDL-C composition even shifts toward a pro-inflammatory phenotype [14]. Low HDL-C levels symbolize dyslipidemia in PD patients and have been attributed to cardiovascular disease (CVD) and mortality in numerous trials [15, 16]. Since CRP may reflect the degree of inflammation and HDL-C may reflect the organism’s anti-inflammatory effect. The combination allows for a comprehensive assessment of the organism’s balance of inflammatory and anti-inflammatory. A high CRP/HDL-C ratio was a potential indicator of poor patient prognosis and increased mortality in a study of patients with novel coronavirus pneumonia [17].

However, there are few scientific studies that combine SII and CRP/HDL-C to predict outcomes in patients with frequent PDAD. As a result, the purpose of this multicenter retrospective study was to investigate the factors associated with the emergence of frequent peritonitis in PD patients treated between 2010 and 2021.

## Materials and methods

### Study subjects

Patients who received regular PD treatment and developed PDAP were recruited for the study and followed up at the Peritoneal Dialysis Center of the Affiliated Hospital of Xuzhou Medical University, Xuzhou Central Hospital, and Xuzhou Mining Group General Hospital between 1/1/ 2010 and 1/9/ 2021. Depending on the number of times peritonitis occurred, patients were divided into two groups: frequent PDAP and non-frequent PDAP. According to ISPD [18, 19], frequent PDAP was defined as the interval between two peritonitis episodes being less than one year. Frequent PDAP includes recurrent peritonitis (an episode that occurs within 4 weeks of completion of therapy of a prior episode but with a different organism), relapsing peritonitis (the episode that occur within 4 weeks of completion of therapy of a prior episode with the same organism or being culture negative), repeat peritonitis (an episode that occurs > 4 weeks after completion of therapy of a prior episode with the same organism).

The inclusion criteria were: (1) Patients with clinically confirmed PDAP; (2) Patients who were older than 18 years old. Exclusion criteria: (1) Patients who died, renal transplantation, and conversion to hemodialysis within one year after the first occurrence of PDAP; (2) Patients with combined hematologic diseases, autoimmune diseases, and malignant tumors; (3) Patients who received PD treatment for less than one month; (4) Patients occurred to peritonitis intervals of less than one month; (5) Patients whose culture bacteria results were fungal infections. The Hospital Committee of Xuzhou Medical University approved this study in 2022. (Ethical number: XYFY2022-KL218)

### PDAP diagnostic criteria and efficacy judgement

All patients received CAPD with lactate-buffered glucose dialysate via a Tenckhoff (Baxter International Inc) peritoneal dialysis catheter with a double bag connection system. All patients attended regularly scheduled PD training sessions led by qualified nursing professionals. In accordance with the 2016 ISPD recommendations, peritonitis was identified. More than two characteristics had to be present for PDAP to be diagnosed [19]: (1) abdominal pain or cloudy dialysate effluent; (2) dialysate WBC counts (WBCs) > 100/µl (after more than two hours of lingering), with polymorphonuclear of > 50%; (3) dialysate effluent culture that is positive.

Efficacy Judgement: (a) Effective Treatment: After three days of antibiotic medication, the symptoms of abdominal pain are eased. In the subsequent laboratory results, the effusion fluid’s turbidity is resolved, and its leukocyte count is either decreased or normalized; (b) Ineffective extubation: When PDAP therapy is unsuccessful, or the patient’s symptoms aggravate as a result of therapy, the PD is extubated and converted to hemodialysis or renal transplant. Due to peritoneal failure or a hernia that cannot be repaired well occurred during therapy; (c) Peritonitis-related death: Patients died from active peritonitis or hospitalization due to peritonitis or died within two weeks of the onset of peritonitis.

### Basic demographic, clinical, and laboratory data

1. Demographic characteristics: baseline data collected at the first PDAP episode. Data were collected when patients were first admitted to the hospital and were not receiving treatment. The information comprised the following: age, gender, comorbidities, history of hypertension (HD), history of diabetes mellitus (DM), cause of ESRD, PD duration, and results of pathogenic bacteria culture.
2. Laboratory parameters: gather information before applying antibiotics. The data included white blood cell counts (WBC), neutrophil (NE), lymphocyte (LY), red blood cell counts (RBC), platelet (PLT), hemoglobin (HGB), red blood cell distribution width (RDW), C-reactive protein (CRP), serum albumin (ALB), serum creatinine (Cr), serum uric acid (UA), blood urea nitrogen(BUN),high-density lipoprotein (HDL-C), low-density lipoprotein (LDL), total cholesterol (TC), triglycerides (TG), serum calcium (Ca), serum phosphorus (P), serum magnesium (Mg), serum potassium (K), glucose (Glu).When suspected peritonitis, dialysate is gathered for dialysate leukocyte count, dialysate polymorphonuclear cells and pathogenic microorganisms.

### Treatment

Dialysate is gathered for cell counting, Gram staining, and bacterial culture when peritonitis is suspected. At the bedside, 8 to 10 mL of dialysate is collected in two blood culture flasks (aerobic and anaerobic), and if there is no positive signal within 48 h, the culture may be extended to five days; another approximately 50 ml of dialysate is collected in sterile tubes for Gram staining and blood agar culture, agar plate culture may be continued under aerobic and anaerobic conditions for three days until microorganisms are detected. According to ISPD Guidelines, the current treatment are vancomycin or first-generation cephalosporin for Gram-positive organism coverage, and third-generation cephalosporin or aminoglycoside for Gram-negative organism coverage by intraperitoneal perfusion in combination [19].

### Definition of SII

SII = platelet count × neutrophils/lymphocytes, NLR = neutrophil/lymphocyte ratio, and PLR = platelet/lymphocyte count, CRP/HDL-C = CRP/HDL-C ratio [9–11].

### Study endpoint

Until they were removed from PD therapy or lost to follow-up owing to conversion to hemodialysis, kidney transplantation, death, etc., patients in this study group were constantly followed. Until September 1, 2022, patients who did not have any of these occurrences were monitored.

## Statistical analysis

For the statistical analysis, SPSS 26.0 software was utilized. The normal distribution of data was expressed as x ± s, and the t-test was performed to distinguish between two groups; if the data did not follow the normal distribution, they were described as M (Q1, Q3), and a non-parametric test was utilized to contrast the groups. The rate of use was applied to represent the statistical data, and the χ² test was implemented to compare the groups.

The relationship between SII and CRP/HDL-C and the risk of developing frequent PDAP was discovered using logistic regression analysis. The ROC curve was used to assess the predictive value of SII and CRP/HDL-C for the development of frequent PDAP in PD patients. A statistically significant difference was regarded as *P* < 0.05.

## Results

**Fig. 1:**
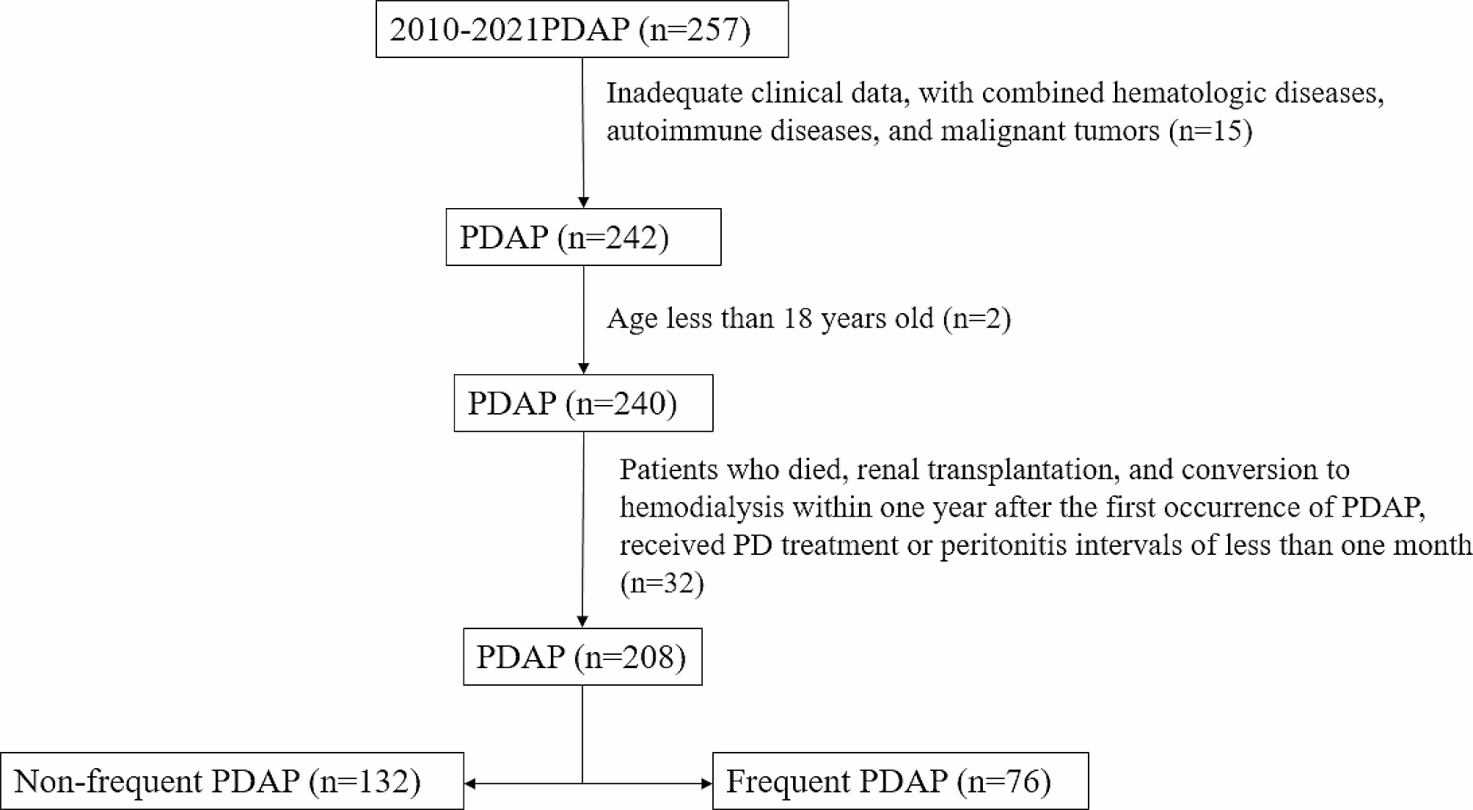
Flow diagram of the study population

Flow diagram of the study population and comparison of the basic data of the two groups: In total, 208 patients with PDAP were recruited from three PD institutions for our study. There were 132 (63.5%) patients in the non-frequent PDAP group and 76 (36.5%) patients in the frequent PDAP group. The average age was 50.5 years, and 126 (60.6%) patients were male. A total of 100 patients (48.08%) had chronic glomerulonephritis, 46 had diabetic nephropathy, 37 (17.79%) had hypertensive nephropathy, and 25 (12.02%) had other diseases. Only the differences in dialysis time (*p* = 0.006), hypertensive nephropathy(*p* = 0.038), and *staphylococcus* (*p* = 0.035) were statistically significant when the baseline data of the two groups were compared. Gram-positive bacterial infections, particularly those caused by *Staphylococcus aureus*, were significantly more likely in the patients who had experienced more episodes. In other organisms, no distinction between the two groups could be discerned (*P* > 0.05) (Table 1) & (Fig. 1).

Laboratory data comparison between two groups: as shown in Table 2, there were statistically significant differences in WBC (*p* = 0.001), NE (*p* < 0.01), LY (*p* < 0.01), PLT (*p* = 0.01), SII (*p* < 0.01), CRP/HDL-C (*p* = 0.002), CRP (*p* < 0.001), Cr (*p* = 0.007), BUN (*p* = 0.05), Mg (*p* = 0.03), K (*p* = 0.007), and dialysate polymorphonuclear cells (*p* = 0.004) between the two groups. There was no difference between age, sex, hypertension, RBC, HGB, RDW, TC, TG, LDL, HDL-C, Ca, P, Glu, and dialysate leukocytes (*P* > 0.05) (Table 2).

**Fig. 2:**
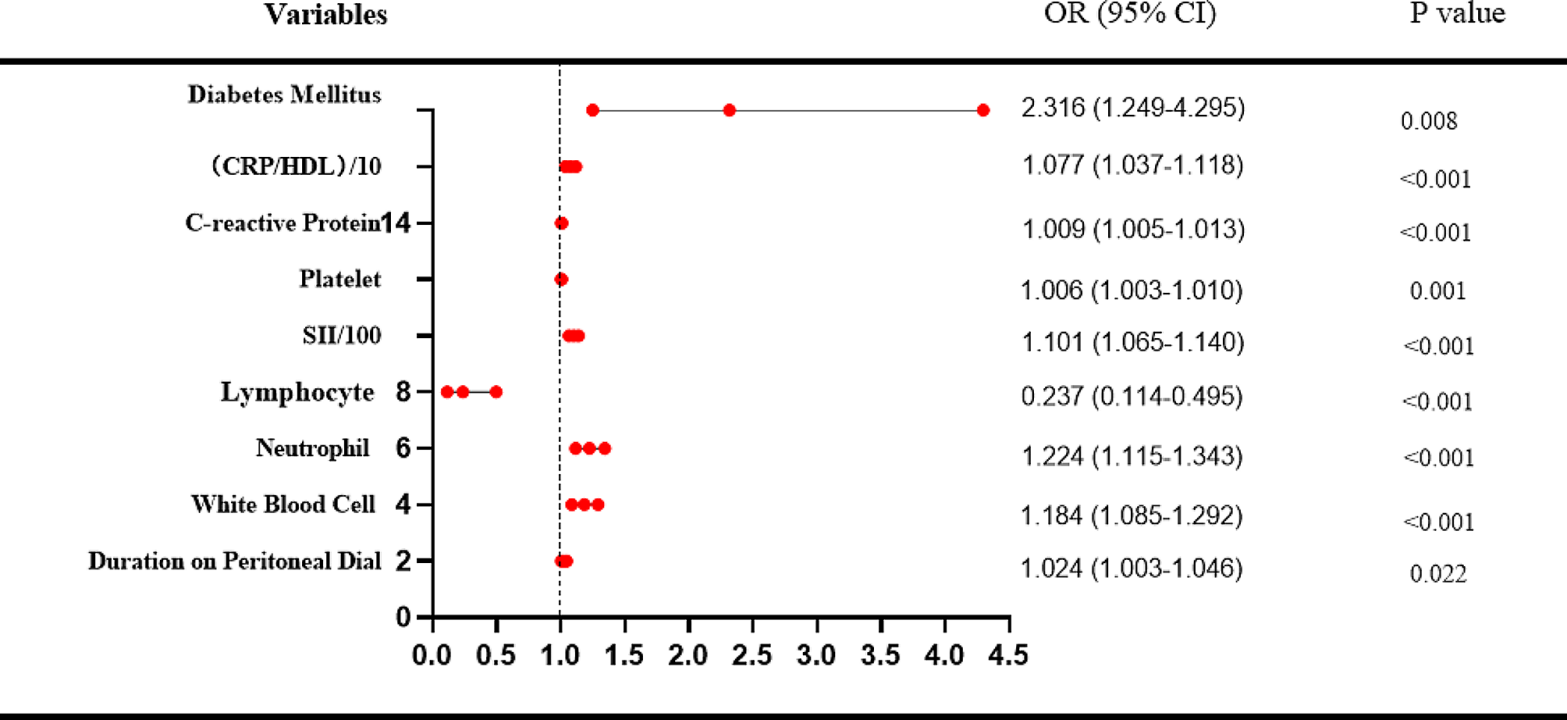
Univariate logistic regression analysis of risk factors in patients with frequent PDAP

Logistic Regression Analysis of Risk Factors in Patients with frequent PDAP: Univariate regression analyses revealed that diabetes mellitus, CRP/HDL-C, CRP, PLT, SII, LY, NE, WBC and PD duration were associated with frequent PDAP. When potential variables such as PD duration, diabetes, CRP/HDL-C, and SII were included, the multivariate stepwise regression analysis revealed that SII (β: 0.001; 95% CI:1.001 to 1.001; *p* < 0.001), CRP/HDL-C (β:0.004; 95% CI: 1 to 1.009; *p* = 0.041) and Diabetes mellitus (β:0.823; 95%CI: 1.1 to 4.72; *p* = 0.027) were independent variables related to frequent PDAP. (Table 3) & (Fig. 2).

**Fig. 3:**
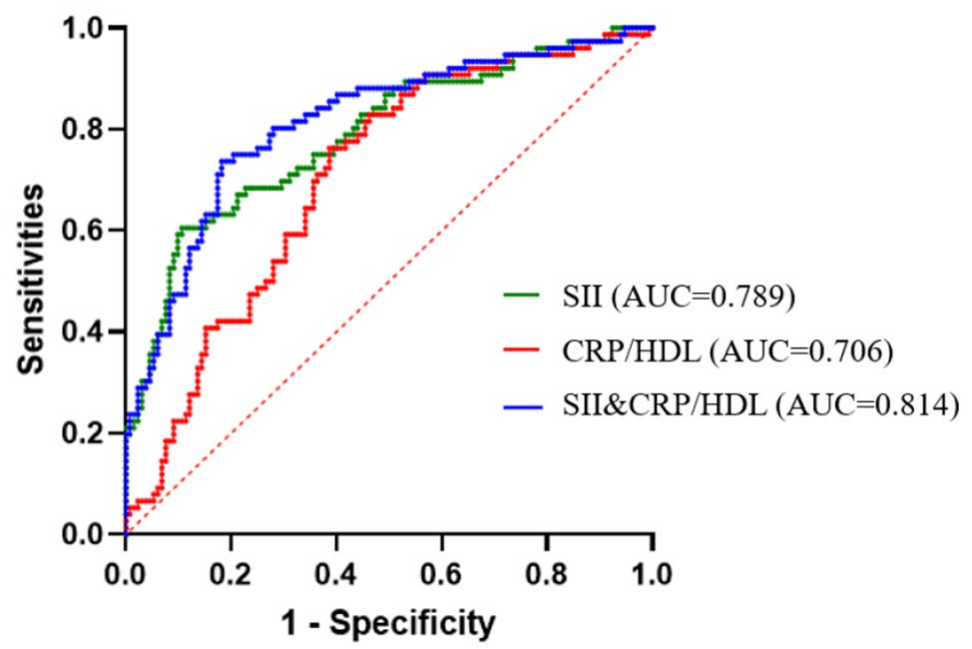
ROC curves of SII, CRP/HDL-C, and SII combined with CRP/HDL-C for predicting frequent PDAP

ROC curves of SII, CRP/HDL-C, and SII combined with CRP/HDL-C for predicting frequent PDAP: excellent discrimination is indicated by the prognosis models, which have an area under the curve (AUC) of over 0.80. The discriminative ability of SII (AUC = 0.789, 95%CI:0.723 to 0.855, *P* < 0.001) and CRP/HDL-C (AUC = 0.706, 95%CI: 0.634 to 0.777, *P* < 0.001) was excellent. The most incredible AUC area was found when SII and CRP/HDL-C were combined (AUC = 0.814, 95%CI: 0.752 to 0.876, *P* < 0.001) (Fig. 3).

**Table 1:** Baseline characteristics of the study population

	non-frequent PDAP	frequent PDAP	t/x	*p*
Gender			2.204	NS
Male	85 (64.39%)	41 (53.94%)		
Female	47 (35.61%)	35 (46.05%)		
Age (year)	51.8 ± 13.34	50.37 ± 13.82	0.737	NS
Etiology of ESRD				
Chronic glomerulonephritis	61 (46.21%)	39 (51.32%)	0.503	NS
Diabetic nephropathy	24 (18.18%)	22 (28.95%)	3.245	NS
Hypertensive nephropathy	29 (21.97%)	8 (10.53%)	4.319	0.038
Other	18 (13.64%)	7 (9.21%)	1.352	NS
Comorbidities				
Hypertension (n, %)	106 (80.30%)	56 (73.68%)	2.43	NS
Diabetes mellitus (n, %)	29 (21.97%)	30 (39.47%)	7.273	0.007
Causal factors [cases (%)]			2.769	NS
mishandling	64	28		
Substandard environment	12	13		
Intestinal infections	46	29		
Exit Tunnel Infection	10	6		
Causative organisms				
Gram-positive (n, %)				
*Staphylococci*	38 (28.79%)	12 (15.79%)	4.463	0.035
*Streptococcus*	11 (8.33%)	8 (10.53%)	0.279	NS
*Enterococcus spp*	3 (2.27%)	1 (1.32%)	0.234	NS
Other	4 (3.03%)	4 (5.26%)	0.65	NS
Gram-negative (n, %)				
*Pseudomonas aeruginosa*	3 (2.27%)	2 (2.63%)	0.026	NS
*Escherichia coli*	26 (19.70%)	14 (18.42%)	0.051	NS
*Klebsiella spp*	3 (2.27%)	2 (5.26%)	0.026	NS
Other	4 (3.03%)	4 (5.26%)	0.65	NS
Culture negative (n, %)	40 (30.30%)	29 (38.16%)	1.324	NS
Outcome				
Effective Treatment	102 (77.27%)	42 (55.26%)	10.968	0.001
Ineffective extubation	20 (15.15%)	21 (27.63%)	4.747	0.029
Peritonitis-related death	10 (7.58%)	13 (17.11%)	4.453	0.035
Follow-up duration	55 (30.5–81.5)	67 (50.5–79)	-1.87	NS

**Table 2:** Comparative analysis of clinical data in two groups

	non-frequent PDAP		frequent PDAP			t/Z/x	*p*
duration on PD(month)	9(3-20.75)		16(6–28)			-2.731	0.006
WBC (10^9/L)	7.35 (6.01–9.23)		9.40 (6.65–12.05)			-3.349	0.001
NE (10^9/L)	5.61 (4.23–7.19)		7.70 (5.27–10.48)			-4.333	< 0.001
LY (10^9/L)	1.07 (0.80–1.30)		0.76 (0.50–1.08)			-4.406	< 0.001
PLT (10^9/L)	195.4 ± 65.32		234.61 ± 95.20			-3.184	0.002
SII	1034.66 (664.64-1522.02)		2379.06 (1235.78-3802.31)			-6.931	< 0.001
MLR	0.39 (0.28–0.54)		0.65 (0.40–0.90)			-4.669	< 0.001
NLR	5.55 (3.77–8.07)		10.43 (6.34–16.85)			-6.237	< 0.001
RBC (10^12/L)	3.21 (2.72–3.75)		3.28 (2.85-4.00)			-1.078	NS
HGB	96.67 ± 20.75		102.41 ± 24.87			-1.787	NS
PLR	185.06 (131.68–241.70)		303.64 (199.58-453.67)			-5.79	< 0.001
RDW (%)	13.70 (13.10–14.60)		13.80 (13.20–14.70)			-0.663	NS
CRP	56.35(16.18-108.98)		121.25(69.4-172.75)			-5.08	< 0.001
ALB (g/L)	32.51 ± 5.57		31.14 ± 6.91			1.467	NS
TC (mmol/L)	4.12 (3.48–5.02)		4.09 (3.46–5.23)			-0.167	NS
TG (mmol/L)	1.10 (0.83–1.58)		1.10 (0.78–1.65)			-0.266	NS
LDL (mmol/L)	2.37 (2.01–3.08)		2.22 (1.83–2.90)			-1.555	NS
HDL-C (mmol/L)	1.19 (0.9–1.48)		1.15 (0.88–1.45)			-0.807	NS
CRP/HDL-C	47.07 (13.83–103.20)		99.45 (65.61-155.95)			-4.938	< 0.001
Cr (umol/L)	825 (607.25-1052.25)		705 (500–931)			-2.714	0.007
BUN (mmol/L)	19.05 (15.4-25.24)		16.68 (12.55–22.49)			-1.958	0.05
UA (mmol/L)	343.5(299.5-404.25)		318(268–399)			-1.965	0.049
Calcium (mmol/L)	2.07 ± 0.23		2.09 ± 0.25		-0.581		NS
Phosphorus (mmol/L)	1.39 (1.13–1.66)		1.29 (1.09–1.52)			-1.524	NS
Magnesium (mmol/L)	0.83 (0.73–0.92)		0.77 (0.68–0.85)			-2.999	0.003
Potassium (mmol/L)	3.94 (3.60–4.62)		3.7 (3.22–4.21)			-2.678	0.007
Glucose (mmol/L)	6.09 (4.90–7.49)		5.88 (5.05–7.90)			-0.314	NS
dialysate leukocyte count (10^6/L)	1929 (674.50-6332.25)		2763 (947.50–5312)			-1.023	NS
dialysate polymorphonuclear cells	82.75 (65.60–90.10)		87.20 (79.75–93.50)			-2.91	0.004

**Table 3:** Multivariate logistic regression analysis of risk factors in patients with frequent PDAP

	B	S.E.	Wald	*p*	OR(95%CI)
CRP/HDL-C	0.004	0.002	4.196	0.041	1.004 (1-1.009)
SII	0.001	0	24.242	< 0.001	1.001 (1.001–1.001)
Duration on PD(month)	0.012	0.013	0.865	0.352	1.012 (0.987–1.037)
DM	0.823	0.372	4.907	0.027	2.278 (1.1–4.72)

## Discussion

PDAP has been one of the most prevalent and severe complications in PD patients, despite variations between nations, regions, and centers of various sizes and levels of care. A prospective cohort analysis conducted between 2014 and 2017 in seven countries revealed that the incidence of PDAP varied between 0.26 and 0.40 episodes/patient-year [20]. Based on our research, SII and CRP/HDL-C appear to have been independent risk factors for frequent PDAP in PD patients. In contrast to high SII levels and low CRP/HDL-C levels alone, high SII levels and concurrently low CRP/HDL-C levels were more strongly related to frequent PDAP in PD patients in our study. Inflammation is a permanent state in PD patients. It is attributed to several factors: microenvironment of uremia, infection, declined removal of pro-inflammatory cytokines, volume overload, oxidative stress, and some dialysis-related variables [10, 21–24]. The reasons for the poor prognosis of patients with healed peritonitis may be related to the presence of chronic inflammation (both systemic and localized in the abdominal cavity) in the patient [25]. The mechanism by which systemic inflammation is mainly related to the release of large amounts of inflammatory mediators causing a decrease in appetite and protein-energy depletion in patients. Although acute infections caused by pathogenic microorganisms resolve, systemic inflammation may persist, leading to frequent episodes of peritonitis as a result of the patient’s compromised immune system and inability to effectively fight off bacterial infection [26]. Localized inflammation in the abdominal cavity is manifested by a persistent increase in inflammatory cells and pro-inflammatory factors in the peritoneal fluid after the peritonitis has resolved [27]. The persistence of local inflammation in the peritoneal cavity can lead to peritoneal mesothelial degeneration, detachment and impaired regeneration, peritoneal fibroblast proliferation and extracellular matrix deposition. Secondly peritonitis decreases the ability of peritoneal solute removal and ultrafiltration, leading to impaired peritoneal structure and function [28].

In the initial phases of inflammation, neutrophils phagocytose pathogens and particles. They simultaneously release antimicrobial peptides and reactive oxygen and nitrogen species [29]. During inflammation, activated platelet count mix with monocytes and lymphocytes to create microparticles that promote the formation of neutrophil extracellular traps (NTEs). NTEs exacerbate the inflammatory response by stimulating the production of inflammatory factors [30]. By encouraging microthrombus, activated platelet count exacerbate the inflammatory response [31]. However, none of these biomarkers provides a comprehensive picture of the body’s inflammatory and immunological condition. SII provides an integrated view of inflammation, immunity, and microthrombus. It has been widely reported that this index can predict the frequency of contrast-induced acute kidney injury (CI-AKI), cancer survival, and cardiovascular disease [32–36]. In 2014, Bo [37] first defined SII as a new indicator of immune evaluation based on a study on the prognosis of patients with hepatocellular carcinoma, and higher SII indicates that patients have stronger inflammation and weaker immune response, suggesting a poor prognosis. The frequent PDAP patients in our study had higher SII scores, which indicated more severe inflammation and a weakened immune system. Increased neutrophil levels frequently indicate an infectious condition and an organism invasion by a specific pathogenic microorganism. The prediction of frequent PDAP is connected with a chronic, long-term, persistent microinflammatory state in patients after the initial infection since it does not depend on the degree of direct invasive overt infection, according to standardized anti-infective therapy for primary PDAP. Thus, this study demonstrates that high SII levels are individually bound to an elevated risk of frequent PDAP after accounting for the duration of PD and DM events. Increased neutrophil-to-lymphocyte ratio (NLR) levels have been exposed to a higher risk of PDAP treatment failure. NLR can also independently predict the severity of cardiovascular disease and mortality [9, 38]. According to earlier studies, a higher SII was tied to a more serious risk of patient death in critically sick patients with acute kidney damage [10].

Meanwhile, this study discovered that patients with frequent PDAP had lower HDL-C concentrations and higher CRP concentrations. The prognostic significance of CRP/HDL-C for frequent PDAP is demonstrated by our findings. HDL-C performs a significant anti-infective function in a variety of methods. Gram-negative bacterial lipopolysaccharide (LPS) and Gram-positive bacterial lip lactic acid (LTA) is bonded and neutralized by HDL-C. HDL-C suppresses the expression of pro-inflammatory cytokine-induced adhesion molecules such as ICAM-1, V-CAM-1, and E-selectin. Additionally, HDL-C blocks the activation and recruitment of monocytes. Reducing ROS generation and inhibiting LDL oxidation offers protection from oxidation [39–40]. HDL-C function was altered in studies comparing CKD patients to the general population, patients with CKD3 and CKD4 stages, and the population receiving hemodialysis. The study reveals that HDL-C mainly affects the apoptosis of polymorphonuclear leukocytes (PMNLs) and the expression of CD11b, leading to a systemic inflammatory response in ESRD patients [41]. Low HDL-C levels result in a reduction in antioxidation, which exacerbates the damage of infection [42]. Low HDL-C levels alone were insufficient to be an independent risk factor for frequent PDAP in our study. This can be since dyslipidemia in PD patients is a complicated process. Disorders of TG, CHOL, and LDL-C are a part of the pathological process of PDAP. The linkage between dyslipidemia and PD is fraught with controversy and uncertainty. As a result, more investigation is necessary to determine how dyslipidemia impacts the prognosis of peritonitis. C-reactive protein (CRP) is a sharp temporal reactive protein compounded by hepatocytes. A persistent increase in CRP indicates that the organism may be experiencing chronic inflammation [43]. Previous research has demonstrated that CRP can be influential in predicting cardiovascular mortality in PD patients [13]. According to retrospective research involving 402 PD patients, for every 1 mg/L increase in ultrasensitive CRP levels, the chance of mortality increased by 1.4% [44]. However, infection, nutritional status, and metabolic health all impact CRP. Therefore, the predictive value of CRP in PDAP patients is debatable. Inflammation severity and anti-inflammatory factors are represented by HDL-C and CRP. The balance of anti-inflammatory and pro-inflammatory factors in the body can be reflected by CRP/HDL-C. In contrast to HDL-C and CRP alone, it can more accurately depict the overall degree of inflammation in PD patients. HDL-C/CRP has been shown to reflect the severity of chronic kidney disease and to be predictive of its progression [45]. No research has examined the correlation between SII, CRP/HDL-C, and frequent PDAP with sizable sample sizes. Therefore, massive sample sizes and longer follow-up dates are still required to validate the study’s findings further.

Our research finds that diabetes mellitus increased the incidence of frequent PDAP (*p* = 0.022), although it was not an independent risk factor. DM has been reported to impair the immunity of peritoneal defenses, such as leukocyte adhesion, chemotaxis, and phagocytosis. Additionally, DM prevents phagocytes from entering the peritoneal cavity and decreases the phagocytic activity of peritoneal macrophages already there [46]. Diabetes increased all-cause mortality in PDAP patients, according to the multicenter retrospective cohort study [47]. According to several pieces of research, DM and low HDL-C levels enhance the likelihood that PDAP may occur [39]. Additionally, some PD patients with diabetes and diabetic retinopathy have an improper peritoneal fluid exchange. The procedure could fail as a result of manipulation problems caused by this. According to our research, the organisms that cause PDAP are more frequently distributed by Gram-positive bacteria. A study found that the *Staphylococcus* epidermidis that causes PDAP has weak immunogenicity, making it simpler to spread infection because the immune system finds it difficult to recognize it [48]. *Staphylococcus* epidermidis is mainly seen in contact with contaminated cases, and the finding that the rate of *staphylococcal* infection in patients in the frequent PDAP group was lower may be related to the fact that the center has strengthened the education on PD care recently, improved the quality of training in aseptic technique for patients, and paid more attention to the prevention of PDAP and optimized the follow-up system.

Limitations of this study are worth noting: (1) Because the study was designed as an observational study, we could not establish a causal relationship between SII, CRP/HDL-C levels, and the likelihood of frequent PDAP events. Additionally, we were unable to rule out the possibility of residual confounding. (2) Because the statistical analysis was based on a single laboratory parameter measurement, it is not possible that the association to remain stable over time. (3) This trial did not specifically document the precise number of episodes of peritonitis in patients with frequent PDAP during a year. (4) This experiment does not further explore the potential reasons of this event.

## Conclusions

In conclusion, our research indicates that SII and CRP/HDL-C are independently attributed to a higher risk of frequent PDAP. SII and CRP/HDL-C are practical and affordable parameters that could serve as novel indicators for patients with repeated episodes of peritonitis. This finding needs further studies to elucidate the potential mechanisms linking SII and CRP/HDL-C to frequent PDAP.

## Data Availability

The data analyzed during this study are included in this published article.

## Abbreviations

CKD: Chronic kidney disease
ESRD: end-stage renal disease
PDAP: peritoneal dialysis-associated peritonitis
LDL: low-density lipoprotein
HDL: high-density lipoprotein
PTH: parathyroid hormone
SII: platelet count × neutrophils/lymphocytes
NLR: neutrophil/lymphocyte ratio
PLR: platelet/lymphocyte count
CRP: C-reactive protein
